# Biochemical and Anatomical Investigation of *Sesbania herbacea* (Mill.) McVaugh Nodules Grown under Flooded and Non-Flooded Conditions

**DOI:** 10.3390/ijms20081824

**Published:** 2019-04-12

**Authors:** Hari B. Krishnan, Nathan W. Oehrle, Alaa A. Alaswad, William (Gene) Stevens, K. M. Maria John, Devanand L. Luthria, Savithiry S. Natarajan

**Affiliations:** 1 Plant Genetics Research Unit, USDA-ARS, Columbia, MO 65211, USA; nathan.oehrle@ars.usda.gov; 2 Plant Science Division, University of Missouri, Columbia, MO 65211, USA; alaaalaswad@mail.missouri.edu; 3 Plant Science Division, University of Missouri, Delta Center, Portageville, MO 63873, USA; stevensw@missouri.edu; 4 Food Composition and Methods Development Laboratory, BHNRC, USDA-ARS, Beltsville, MD 20705, USA; mariajohn79@gmail.com (K.M.M.J.); dave.luthria@ars.usda.gov (D.L.L.); 5 Soybean Genomics and Improvement Laboratory, USDA-ARS, Beltsville, MD 20705, USA; savi.natarajan@ars.usda.gov

**Keywords:** Sesbania, waterlogging, nodulation, nitrogen fixation, aspartate aminotransferase

## Abstract

*Sesbania herbacea*, a native North American fast-growing legume, thrives in wet and waterlogged conditions. This legume enters into symbiotic association with rhizobia, resulting in the formation of nitrogen-fixing nodules on the roots. A flooding-induced anaerobic environment imposes a challenge for the survival of rhizobia and negatively impacts nodulation. Very little information is available on how *S. herbacea* is able to thrive and efficiently fix N_2_ in flooded conditions. In this study, we found that Sesbania plants grown under flooded conditions were significantly taller, produced more biomass, and formed more nodules when compared to plants grown on dry land. Transmission electron microscopy of Sesbania nodules revealed bacteroids from flooded nodules contained prominent polyhydroxybutyrate crystals, which were absent in non-flooded nodules. Gas and ion chromatography mass spectrometry analysis of nodule metabolites revealed a marked decrease in asparagine and an increase in the levels of gamma aminobutyric acid in flooded nodules. 2-D gel electrophoresis of nodule bacteroid proteins revealed flooding-induced changes in their protein profiles. Several of the bacteroid proteins that were prominent in flooded nodules were identified by mass spectrometry to be members of the ABC transporter family. The activities of several key enzymes involved in nitrogen metabolism was altered in Sesbania flooded nodules. Aspartate aminotransferase (AspAT), an enzyme with a vital role in the assimilation of reduced nitrogen, was dramatically elevated in flooded nodules. The results of our study highlight the potential of *S. herbacea* as a green manure and sheds light on the morphological, structural, and biochemical adaptations that enable *S. herbacea* to thrive and efficiently fix N_2_ in flooded conditions.

## 1. Introduction

*Sesbania herbacea* (Mill.) McVaugh is commonly known as bigpod sesbania, Colorado river-hemp, and coffeeweed. *Sesbania herbacea* is a native to North America. This plant thrives in highly disturbed habitats, sandy sites, shallow flooded areas, and cultivated fields. In the United States, the distribution of *S. herbacea* ranges from New York to the Southeast, and southwest to Texas and California [1]. It grows extremely well in the alluvial clay soils of the lower Mississippi River Valley [2]. In addition, it is also found in Mexico and Central America. *S. herbaceae* is considered to be an invasive weed and, if left uncontrolled, can reduce yields of cash crops such as soybean, cotton, and rice [3,4,5,6]. 

*Sesbania herbacea* is a fast-growing nodulating legume that can produce 100–146 kg·ha^−1^ N in above ground biomass [7]. It is a prolific seed producer yielding up to 21,000 seeds per plant [8]. Grown mainly as a soil-improving crop [9], it was once used extensively as a cover crop in citrus groves [10] and by cotton growers in California [6]. In contrast to most legumes that are sensitive to flooding [11], *S*. *herbacea* is tolerant to flooding and performs well in wet and waterlogged conditions. Water-logged soil represents an anaerobic environment and imposes a challenge for the survival of rhizobia and negatively impacts nodulation [12,13,14]. Flooding results in low O_2_ status in root tissues that could negatively impact the legume-rhizobia symbioses [15]. Biological nitrogen fixation requires a high proportion of ATP (16 molecules of ATP for every molecule of NH_3_) which is generated during respiratory electron transport reactions. Nitrogenase, the enzyme responsible for the reduction of nitrogen (N_2_) to ammonia (NH_3_), is sensitive to O_2_ levels. Under flooded conditions, most terrestrial legumes are not capable of increasing the O_2_ supply and hence are not able to fix atmospheric nitrogen effectively. Legumes occurring in wetlands have evolved morphological, structural, and biochemical adaptations that enable them the overcome the deleterious effect of flooding [11]. 

The genus *Sesbania* includes about 70 species and forms nitrogen-fixing nodules on the roots, and in the case of *S. rostrata,* nodules are formed both on root and stem [10]. Nodules have been recorded for 27 of the 70 species of *Sesbania* [16]. Extensive studies have been carried out on the symbiotic interaction between *S. rostrata* and *Azorhizobium caulinodans* [17,18,19]. In contrast to this well-studied symbiosis, nodulation of the other members of the genus *Sesbania* have received only limited attention. In Mexican soils, the microsymbiont isolated from the nodules of *S. herbacea* from flooded fields were identified as *Neorhizobium huautlense* [12]. Based on the PCR-RFLP of 16S rRNA genes, RFLP of *nifH*, and *nodDAB* genes, the microsymbiont of *S. herbacea* was reported to be phylogenetically related to *Rhizobium galegae* [20]. Another Sesbania nodulating rhizobium isolated from the Indian soil, *Rhizobium* sp. SIN-1, was also found to be phylogenetically related to *Rhizobium galegae* [21]. *Mesorhizobium sp.*, and fast-growing rhizobia related to *R. tropici* and *R. etli* have also been reported to form nodules on *S. herbacea* [20]. *Sinorhizobium meliloti* and *Rhizobium (Agrobacterium) strain IRBG74* were found to induce ineffective nodules on this legume [22,23]. The focus of most of the previous studies were on the rhizobial symbionts, but very little information is available on the biochemical and anatomical adaptations that enable *Sesbania herbacea* to fix N_2_ in flooded conditions. In this study, we have investigated the biochemical and anatomical changes in the *S. herbacea* nodules grown under flooded and non-flooded conditions. 

## 2. Results

### 2.1. Sesbania Plants Grown in Flooded Fields Produce More Biomass

**Figure 1:**
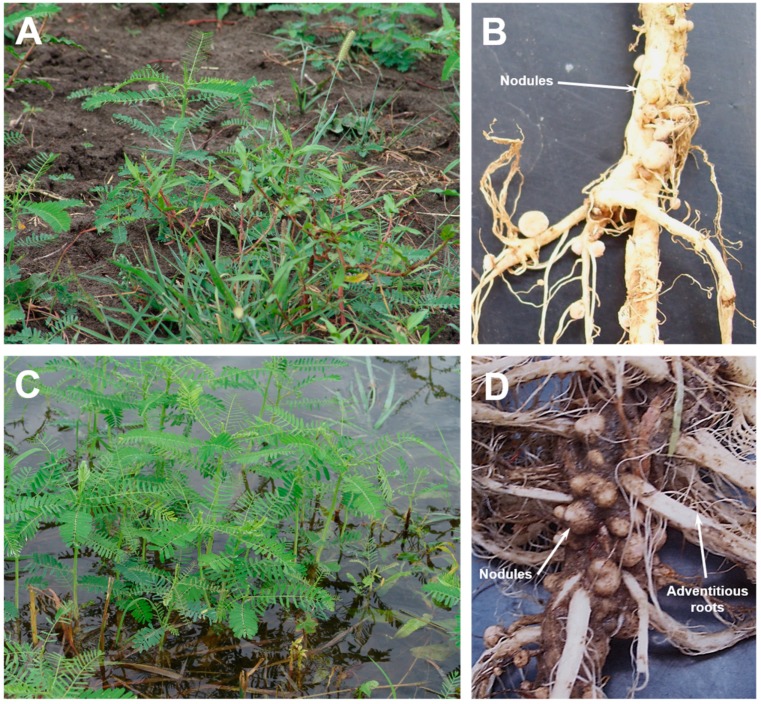
Photographs of *Sesbania herbacea* plants growing in non-flooded (**A**) and flooded (**C**) conditions in field plots at Portageville, MO. Root nodules are found in both non-flooded (**B**) and flooded plants (**D**). Note the presence of numerous nodules and adventitious roots in Sesbania plants growing under flooded conditions. Photographs were taken about 30 days post germination.

In southeast Missouri, *Sesbania herbacea* (referred to as Sesbania from this point onwards) is commonly found in croplands (Figure 1A) and in flooded fields (Figure 1C). This weed possesses green pinnately compound leaves and is capable of growing 1–3 m tall. A comparison of the root system between plants grown in dry land and flood conditions reveal drastic differences. Numerous adventitious and whitish spongy roots were seen in plants that were grown in flooded conditions (Figure 1D). These spongy roots were not observed in dry land plants (Figure 1B). Nodules were observed on the roots of plants grown both in dry and flooded conditions. Nodules from plants grown in non-flooded conditions are mostly spherical, though some indeterminate-type nodules were also observed (Figure 1B). In contrast, the nodules observed on the roots of plants grown in flooded conditions were strikingly large and numerous (Figure 1D). These nodules were clustered on both main and lateral roots and had an apical meristematic region typical of indeterminate-type nodules. Nodules harvested from both dry land and flooded plants revealed a pink interior indicating that these nodules are capable of nitrogen fixation. 

Sesbania grown in the field under flooded and non-flooded conditions differed significantly in several physiological traits (Table 1). Plants grown in flooded conditions were strikingly taller than those plants grown under non-flooded condition. Flooded plants were nearly twice as tall as those grown in dryland. The diameter of the stems from flooded plants were also significantly greater than those from dryland grown plants. Importantly, the biomass of the Sesbania grown in flooded conditions were nearly three times greater than the plants grown in the dryland. In spite of these significant differences in the plant growth, the number of pods produced per plant remained unchanged regardless of whether the plants were grown under flooded or non-flooded conditions. We also measured the total nitrogen/protein content of seeds using an organic elemental nitrogen macro analyzer. This analysis revealed that the protein content of seeds harvested from plants grown under flooded and non-flooded conditions were not statistically different (Table 1). Thus, flooding seems to have no pronounced effect on S. *herbacea* seed production and protein content, but significantly affects overall biomass.

### 2.2. Flooding Increases the Number of Nodules on Sesbania Roots

A striking feature of Sesbania grown under flooded conditions is the presence of massive number of nodules on their roots (Figure 1D). We first isolated the bacteria from field grown nodules and determined their 16S ribosomal RNA sequence. A BLAST analysis of the 16S ribosomal RNA sequence revealed 100% sequence homology to 16S ribosomal RNA of *Neorhizobium huautlense*, a microsymbiont of *S. herbacea* [12,24]. A visual observation of the roots clearly reveals that the flooded Sesbania produced more nodules than those from dry land. However, an exact count on the number of nodules was challenging since it was difficult to pull out the entire root system in the field without damaging the lateral roots. To confirm that Sesbania grown in flooded conditions produce more nodules than dry land grown plants, we performed nodulation assays under flooded and non-flooded conditions in an environmentally controlled growth chamber. Sesbania plants grown in non-flooded conditions had significantly less nodules per plant than those plants grown in flooded condition (Table 1), however the average fresh weight (per nodule) between the non-flooded and flooded treatments were not significantly different. Similarly, as seen in the field experiments, the average height of the plants from flooded treatments were higher. These results demonstrate that flooding causes a marked increase in the number of nodules and promotes plant growth and accumulation of mass, similar to what we observed in field experiments. 

### 2.3. Comparison of Nodule Anatomy of Sesbania Nodules Grown under Flooded and Non-Flooded Conditions

**Figure 2:**
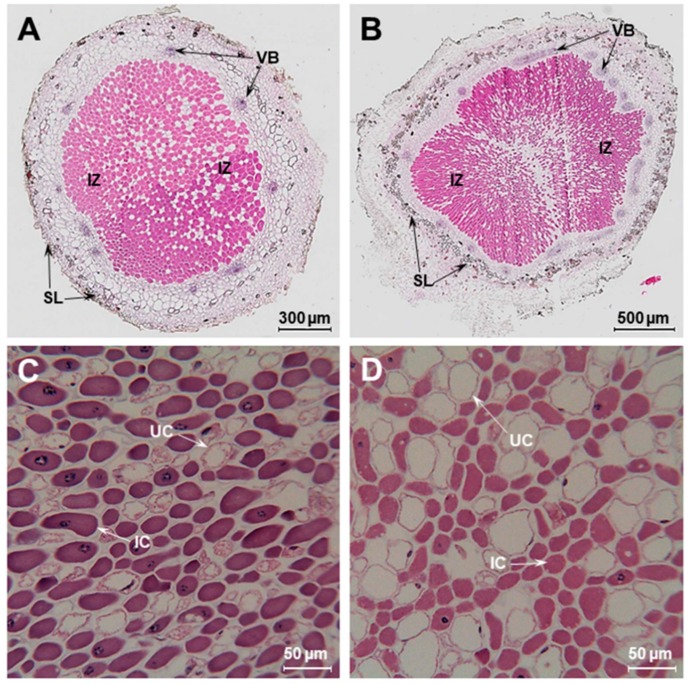
Anatomy of Sesbania nodules. A cross-section of Sesbania nodules growing in non-flooded (**A**) and flooded (**B**) conditions. The infected central region is filled with rhizobia and is surrounded by a scleroid layer. A high magnification of the infected central region reveals the occurrence of both infected and uninfected cells (**C**,**D**). The uninfected cells of flooded nodules (D) are much larger than that of non-flooded nodules (C). IC, infected cells; UC, uninfected cells; VB, vascular bundle; SL, scleroid layer, IZ, infected zone.

Sesbania plants grown under flooded conditions exhibited proliferation of adventitious roots and increased number of nodules (Figure 1D). Numerous studies have established that flooding induces the formation of aerenchyma in the cortex of roots which increases tissue porosity to facilitate O_2_ movement within roots [25,26]. The anatomy of flooded nodules have been described for several wetland legumes, such as *Neptunia* [27,28], Discolobium [29,30], Aeschynomene [30,31], *Lotus uliginosus* [32,33], as well as forage legumes like clover [34] and *Melilotus siculus* [35,36]. We also investigated the effect of flooding on the anatomy of Sesbania nodules that were grown under flooded and non-flooded conditions using light microscopy (Figure 2). Both flooded and non-flooded nodules had a large central region that was occupied by bacteria. The infected zone (IZ) was surrounded by the cortex region that contained prominent vascular bundles. The cortex was surrounded by a scleroid layer that were much more prominent in the flooded nodules compared to non-flooded nodules (Figure 2B). A higher magnification of the infected zone revealed the presence of both infected and uninfected cells. The infected cells were distorted and shrunken in their appearance, presumably due to the harsh paraffin embedding procedure. Interestingly, the infected zone found in the flooded nodules contained uninfected cells that were appreciably larger than those found in the non-flooded nodules (Figure 2D). Transmission electron microscopy observation of Sesbania nodules revealed prominent polyhydroxybutyrate (PHB) granules in bacteroids from flooded Sesbania nodules but not in non-flooded nodules (Supplementary Figure S1). It is known that PHB granules could serve as sinks of excess carbon and reducing power and could be utilized as carbon and energy reserves when bacteria are exposed to oxygen-limiting conditions [37,38,39].

### 2.4. Changes in Sesbania Nodule Metabolites Due to Flooding

**Figure 3:**
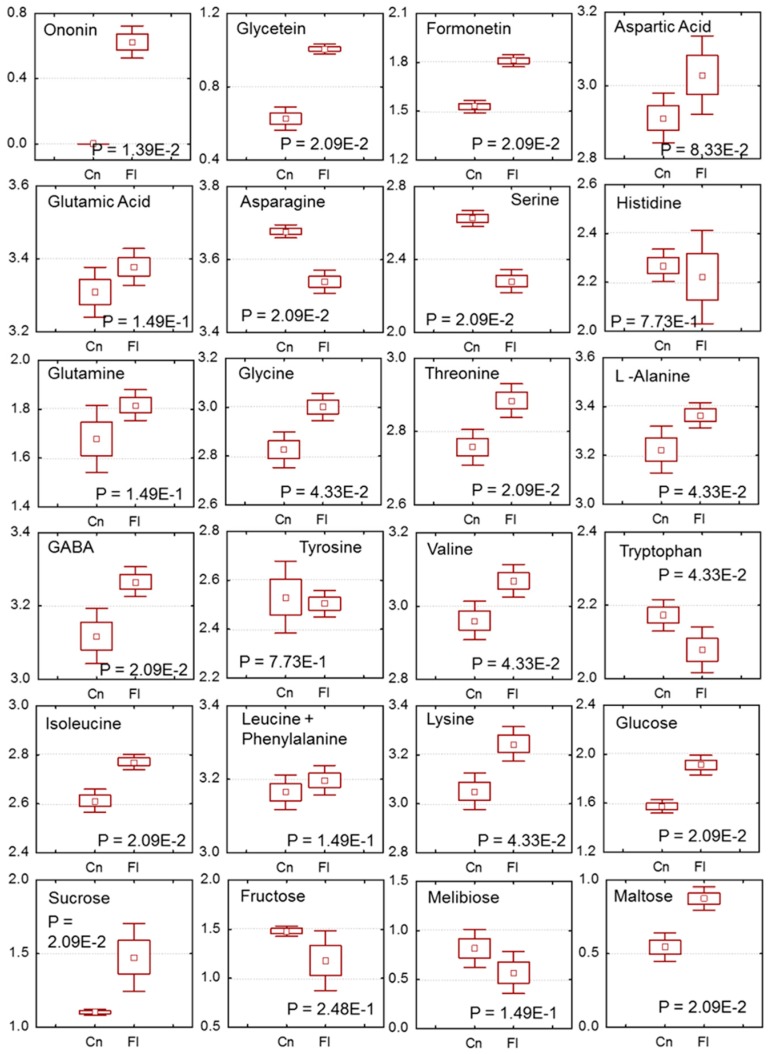
Changes in primary and secondary metabolites in non-flooded and flooded Sesbania nodules. Data were collected using LC-MS and ion chromatography–mass spectrometry (IC-MS). Metabolites were identified based on available standards, mass, and references and grouped into three categories; secondary metabolites, amino acids, and sugars. Results are from three separate extractions, with samples ran in triplicate. Cn, non-flooded; Fl, flooded.

Root waterlogging is known to influence nitrogen metabolism in the nodules and bring about series of metabolic changes that alters carbohydrate, energy, and amino acids levels [40,41,42,43]. However, very little is known if similar changes also occur in Sesbania nodules that are subjected to waterlogging. To investigate metabolic changes in Sesbania nodules, we measured the content of 24 metabolites utilizing LC-MS and ion chromatography–mass spectrometry (IC-MS) (Supplementary Table S1). These metabolites were identified based on available standards, mass, and references and grouped into three categories; secondary metabolites, amino acids, and sugars. A targeted metabolites analysis was prepared using partial least-squares-discriminate analysis (PLS-DA) model to see the difference between the groups along with the metabolites contributing variation between the groups (Supplementary Figure S2). The control and flooded plots showed two different clusters in the scoring plot (Supplementary Figure S1A). The difference between the groups was observed to be ~80% based on PSLC 1 and 2 differences. The loading plot analysis revealed that ononin, glycitein, formonetin, asparagine, serine, lysine, glucose, and maltose highly influenced the clustering pattern (Supplementary Figure S2B). These metabolites were compared with box whisker plot, and the amount of isoflavone glycoside ononin, phytoestrogens formononetin, and glycitein was found elevated in flooded nodules when compared to that non-flooded nodules (Figure 3). A comparison of the amino acid profile of flooded and non-flooded nodules also revealed significant differences. Asparagine and serine occur at relatively higher amounts in non-flooded control nodules, while the levels of glutamine, glycine, threonine, alanine, isoleucine, and lysine were greater in flooded nodules. Interestingly, the levels of gamma aminobutyric acid (GABA), a primary stress indicator in flooded soils, was also found to be higher in flooded nodules when compared to non-flooded nodules (Figure 3). An examination of carbohydrate levels showed that flooding resulted in an increase in the levels of glucose, sucrose, and maltose within nodules.

### 2.5. Flooding-Induced Changes in Enzyme Activities

**Figure 4:**
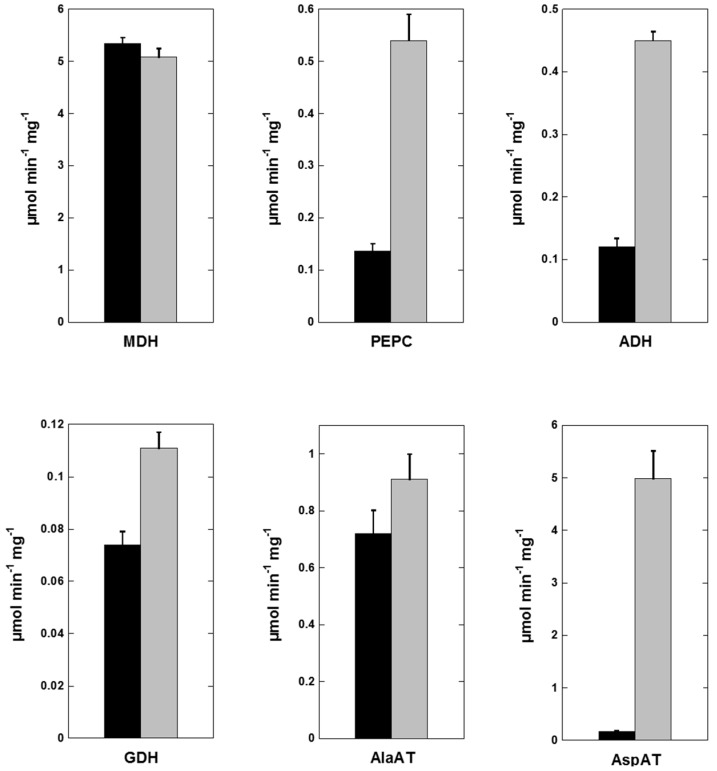
Enzyme activities from non-flooded and flooded Sesbania nodules. Malate dehydrogenase (MDH), phosphoenolpyruvate carboxylase (PEPC), alcohol dehydrogenase (ADH), glutamate dehydrogenase (GDH), alanine aminotransferase (AlaAT), and aspartate aminotransferase (AspAT) activity from non-flooded (black bars) and flooded nodules (gray bars) were measured spectrophotometrically. Average values are shown ± SD (*n* = 3). Enzyme activity is expressed as μmol·min^−1^·mg^−1^ protein.

Flooded plants adapt to waterlogging conditions by initiating changes in critical enzyme activities to alleviate the consequence of oxygen deprivation [44]. To investigate changes in flooded and non-flooded Sesbania nodules, we carried out measurement of several key enzymes involved in nitrogen metabolism. We found no significant differences in the activity of nitrogenase when assayed in whole intact nodules (Table 1). In nitrogen-fixing nodules, malate is a major source of energy for bacteroid respiration and its synthesis is catalyzed by malate dehydrogenase through a reversible reduction of oxaloacetate to malate [45,46]. Malate dehydrogenase (MDH) activity was abundantly present in both non-flooded and flooded nodule plant cytosol (Figure 4) with no significant differences in amount of activity. We also measured the activity of phosphoenolpyruvate carboxylase, an enzyme with an important role in carbon metabolism in symbiotic nodules [47,48]. In contrast to MDH, the activity of phosphoenolpyruvate carboxylase (PEPC) was significantly elevated in flooded Sesbania nodules (Figure 4). Similarly, the activity of alcohol dehydrogenase, a fermentative enzyme that converts acetaldehyde to ethanol, was also elevated several-fold in flooded nodules. Measurement of glutamate dehydrogenase (GDH), an enzyme involved in ammonia assimilation, was also found to be elevated in flooded nodules. 

A common response to flooding is the accumulation of alanine [43,49]. Alanine aminotransferase (AlaAT) catalyzes the reversible reaction between pyruvate and glutamate into alanine and 2-oxoglutarate. Earlier studies have established that AlaAT is induced by waterlogging or flooding [43,44,49]. In this study, we observed AlaAT activity in flooded Sesbania nodules was marginally higher when compared to control nodules. In contrast, the activity of aspartate aminotransferase (AspAT), an enzyme with a vital role in the assimilation of reduced nitrogen, revealed a dramatic change. This enzyme catalyzes the reversible transamination between aspartate and 2-oxoglutarate into glutamate and oxaloacetate [50]. Flooded nodules demonstrated a 14-fold increase in AspAT activity when compared to non-flooded nodules (Figure 4) in the direction of oxaloacetate production.

### 2.6. Western Blot Analysis Reveals a Higher Accumulation of Aspartate Aminotransferase in Flooded Nodules

**Figure 5:**
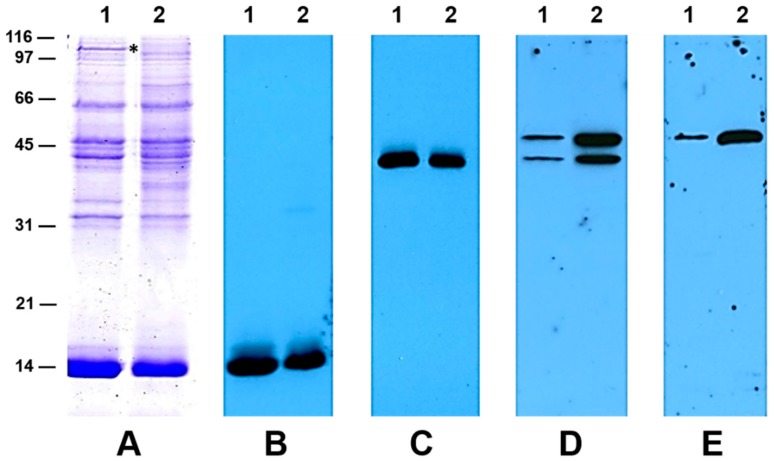
SDS-PAGE and immunoblot analysis of Sesbania nodule proteins. Total protein from non-flooded (Lane 1) and flooded (Lane 2) Sesbania nodules were extracted and separated using 13.5% SDS-PAGE. Resolved proteins were visualized by staining with Coomassie Blue (**A**) and transferred to nitrocellulose and probed with antibodies raised against leghemoglobin (**B**), malate dehydrogenase (**C**), aspartate aminotransferase-1 (**D**), and aspartate aminotransferase-2 (**E**). Protein cross reacting with antibodies were detected using anti-rabbit IgG-horseradish peroxidase conjugate followed by chemiluminescent detection. Molecular weight markers on the left side of figure are in kilodaltons. A unique protein in non-flooded nodules is indicated with an asterisk.

To investigate whether flooding induced protein changes in the nodules, we isolated total proteins from non-flooded and flooded Sesbania nodules and resolved them by SDS-PAGE (Figure 5). The protein profile of both flooded and non-flooded nodules were similar, though differences in the intensity of some polypeptides were evident. Leghemoglobin, the most abundant nodule protein, accumulated slightly at lower levels in flooded nodules. A high molecular weight protein (>100 kDa) was prominently seen in non-flooded nodules but not in the flooded nodule extracts. We also employed antibodies raised against leghemoglobin, malate dehydrogenase, and aspartate aminotransferase to monitor their accumulation in non-flooded and flooded nodules. Western blot analysis revealed that the accumulation of leghemoglobin (Figure 5B) and malate dehydrogenase (Figure 5C) was comparable in both non-flooded and flooded nodules. In contrast, the accumulation of AspAT was drastically higher in flooded Sesbania nodules (Figure 5D,E). In alfalfa, AspAT occurs as two forms, AspAT-1 and AspAT-2 [51]. AspAT-1 (42 kDa) was reported to be abundant in the roots while AspAT-2 (50 kDa) was predominant in nodules. Antibodies raised against AspAT-1 reacted strongly against two Sesbania nodule proteins with apparent molecular weights of 42 kDa and 50 kDa, while AspAT-2 antibodies reacted against a 50 kDa protein only. The accumulation of AspAT was dramatically higher in flooded nodules, which could explain the high level of AspAT activity detected in the flooded nodules.

### 2.7. 2-D SDS-PAGE and Protein Identification by Mass Spectrometry

**Figure 6:**
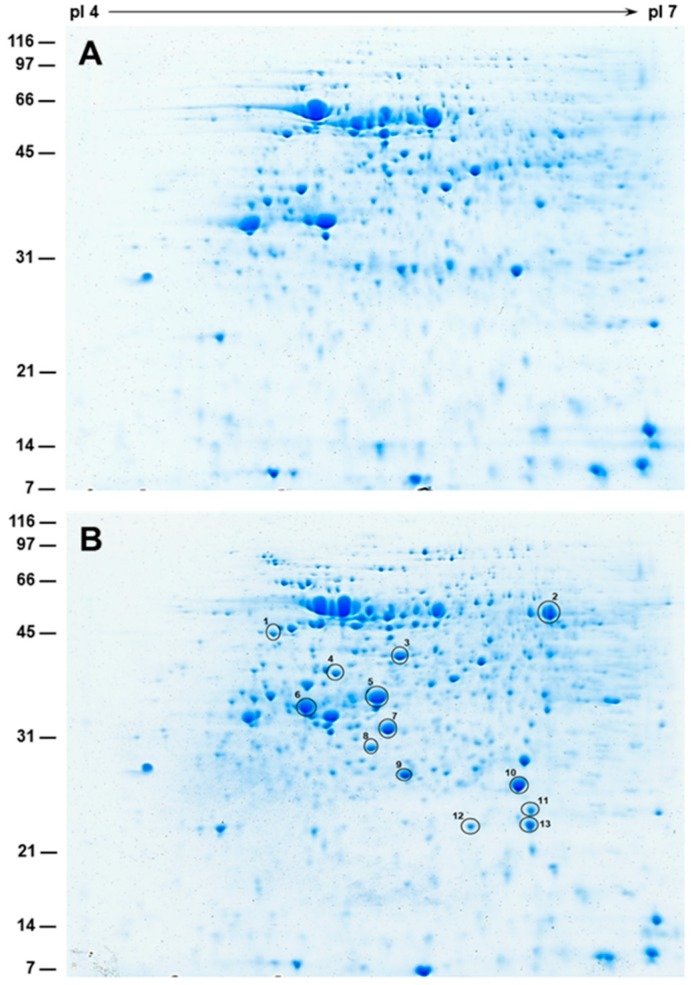
Two-dimensional gel electrophoresis of Sesbania nodule bacteroid proteins. Three hundred micrograms of nodule bacteroid proteins from non-flooded (**A**) and flooded (**B**) were separated by isoelectric focusing on pI 4-7 strips followed by SDS-PAGE on 15% gels. Following electrophoresis, the gels were stained with Colloidal Coomassie Blue G-250. The position and sizes of protein markers in kDa are shown on the left of the figures. Protein spots that were elevated in flooded nodule bacteroids (enclosed in circles) were picked from the gels for identification using matrix-assisted laser desorption ionization-time of flight (MALDI-TOF-MS)/MS analysis. Numbered circles correspond to the # in Table 2.

To detect flooding-induced changes in the protein profiles, we performed one-dimensional gel analysis of nodule cytosol and bacteroids protein fractions. 1-D analysis of nodule cytosol proteins (minus bacteroids) resulted in the visualization of numerous discrete protein bands. A comparison of nodule cytosol protein fractions from non-flooded and flooded nodules revealed similar distribution of protein bands, though differences in the intensity of some of the protein bands were noticeable. Interestingly, a comparison of nodule bacteroid fractions revealed the presence of few unique protein bands in the flooded nodule protein fraction (data not shown). To further differentiate the flooding-induced changes in the bacteroids’ protein fractions, 2-D gels were performed (Figure 6). A comparison of 2-D gels of non-flooded and flooded nodule bacteroid protein fractions revealed several unique protein spots in the flooded nodule bacteroid protein fraction (Figure 6B). To identify some of these protein spots, we individually extracted them, digested them with trypsin, and analyzed them by matrix-assisted laser desorption-ionization time of flight mass spectrometry (Table 2). The peptide sequences generated from this analysis were searched against the National Center for Biotechnology Information non-redundant (NCBInr) microorganism protein database and revealed significant sequence homology to several *Neorhizobium huautlense* proteins (Table 2). Several of the unique proteins identified in the flooded nodule bacteroid fraction belonged to the ABC transport family. ABC transporters are important for efficient nitrogen fixation and have been proposed to function in an amino acid cycle between the bacteroid and the host [52].

## 3. Discussion

Nitrogen-fixing legumes have an important role in enriching N-deficient soils and maintaining the biodiversity of wetland plants [11,53]. Some of the wetland legumes, especially stem-nodulating ones, have been utilized as green manure crops due to their high rates of N_2_ fixation under flooded conditions [18,54]. The results of our study demonstrate that *S. herbacea* is well adapted to flooded conditions and can make a positive contribution to the enrichment of N-deficient soils. Its potential to contribute to N-cycle has been recognized in certain regions of USA where they are grown mainly as a soil-improving crop [9]. Since this crop thrives well in flooded conditions, they have great potential to be exploited as a green manure, especially in the rice-growing regions of Missouri and Arkansas.

Sesbania plants growing under flooded conditions performed better than those grown in dryland. The flooded plants were twice as tall as the control plants and showed a significant increase in their biomass. Previously, it has been shown that the plant hormone ethylene can act as a growth stimulator by promoting stem elongation and adventitious root growth of plants in flooded conditions [55,56]. Thus, the physiological adaptations observed in Sesbania plants could be mediated by ethylene, which accumulates in the rhizosphere of flooded soils. Another striking feature of Sesbania plants grown under flooded conditions is the presence of large number of nodules on the root system. Interestingly, ethylene is known as a potent inhibitor of nodulation since they block cortical cell division and reduce the number of cortical infections [57]. This response may not be universal since legumes that are ethylene-insensitive have also been reported [58,59]. In this regard, it should be pointed out that ethylene is required for nodulation on stems and submerged roots of *S. rostrata* [60]. Similarly, reduced ethylene production by flooded *Lotus uliginosus* was linked with higher nodulation and plant growth under hypoxic flooded conditions [32].

Nodule development in *Sesbania* spp. under non-flooded and flooded conditions have been previously investigated [23,60,61,62]. In *S. rostrata*, the rhizobia enter the host intercellularly, in cracks present at bases of lateral and adventitious roots. The plant hormone ethylene and reactive oxygen species have been shown to be important for the intercellular entry and nodule primordium initiation [60]. In *S. virgate* and *S. cannabina*, the intercellular invasion of rhizobia occurs at the lateral root bases when the plants were under flooded conditions but under non-flooded conditions they occur via root hairs [23,61,62]. Interestingly, under non-flooded conditions, the nodules formed were of determinate type, while under flooded conditions the nodules were of both determinate and indeterminate type. In contrast, in our study, we observed both determinate and indeterminate nodules under both flooded and non-flooded conditions. One possible reason for this discrepancy could be attributed to fact that our observations were based on plants that were grown in fields that were subjected to prolonged flooding and not in environmentally controlled growth chambers where the plants were subjected to flooding for only a limited time.

Waterlogged soils create anoxic conditions, which are a known stress factor for plants. Oxygen deprivation possess a serious problem since plant roots are aerobic. Fortunately, plants have developed morphological and biochemical adaptations that mitigate the harmful effects of oxygen depletion. Flooding induced metabolic changes have been investigated in several studies [40,42,63,64,65]. Under low oxygen concentration, anaerobic metabolism is activated, a decrease in energy production, accumulation of pyruvate and intermediates of Krebs cycle, and a decrease in NAD(P)+ levels are observed [63]. Flooding has been shown to cause a marked decrease in asparagine and an increase in γ-aminobutryic acid (GABA) in soybean nodules [64]. In our metabolite analysis, we also observed a significant decrease in asparagine and an increase in GABA in Sesbania nodules under flooded conditions. This response appears to be specific to nodules since in other tissues, including roots exposed to flooding, alanine accumulation has been reported [40,65]. An increase in GABA and a decrease in asparagine is observed in nodules irrespective of whether the plants were subjected to flooding for a short (eight days, Reference [64]) or long duration (three months, current study). The physiological significance for the increase in GABA in soybean nodules under flooded conditions has been discussed earlier [64]. It is speculated that GABA may play a role in regulation of intercellular pH, protection against oxidative stress, and as a signal molecule [66]. Alternatively, it was proposed that GABA could serve as a temporary nitrogen reserve in flooded nodules [64].

Plants accumulate alanine (Ala) when the oxygen availability decreases during waterlogging or flooding [43,49]. Ala accumulation occurs due to a rapid induction of the alanine aminotransferase (AlaAT) gene expression and an increase in the enzyme activity [67,68,69]. Under anoxic conditions, the primary source of energy is the glycolytic pathway which results in the production of two ATP and two pyruvate molecules per unit of hexose along with the reduction of NAD+ to NADH [44]. It is not clear how the accumulation of Ala will sustain anoxic metabolism. It was proposed that Ala accumulation would help to regulate the pH balance within oxygen-deprived cells [70]. The role of alanine accumulation during waterlogging has been investigated in a highly flood-tolerant nodulated *Lotus japonicus* [44]. Based on this study, a metabolic model was proposed which explains why alanine and succinate accumulate and the production of extra ATP for the continued operation of glycolysis during anoxia [44].

In this study, even though we observed an increase in alanine aminotransferase activity under flooded conditions, it was negligible when compared to that of aspartate aminotransferase activity (AspAT). The drastic increase in AspAT activity suggests a major role for this enzyme in anoxic metabolism. AspAT catalyzes the reversible transamination between glutamate and oxaloacetate into aspartate and 2-oxoglutarate. Several isozymes of AspAT have been reported in legumes with different subcellular locations [50]. AspAT activity in nodules is mostly of plant origin and is involved in regulating C and N flow between microbial symbiont and host plant cytoplasm [51]. AspAT is proposed to act as a shuttle system to maintain a supply of oxaloacetate to bacteria or function as part of a malate-aspartate shuttle. Additionally, AspAT may also be involved in the shuttling of redox equivalents between subcellular and cellular compartments [50]. In our study, an increase in AspAT activity in flooded nodules could lead to an increase in 2-oxoglutarate, which, in turn, can be converted to succinate in a two-step reaction catalyzed by α-ketoglutarate dehydrogenase and succinate CoA ligase. This process will enable the ATP production with concomitant reduction of NAD+ to NADH, resulting in efficient operation of glycolysis in Sesbania nodules under flooded conditions.

An examination of protein changes in Sesbania flooded nodules revealed an increase in the concentration of several bacteroid proteins belonging to the ABC transport family (Table 2). The abundance of these proteins in the flooded nodules suggest that they could play a role in the exchange of metabolites between the bacteroid and the host cells for maintaining efficient nitrogen fixation under flooded conditions. Interestingly, one of the proteins in Sesbania flooded nodules that shows an increase in concentration has been identified as nitrogenase. Leghemoglobin, an O_2_-carrying protein, is critical for transporting dissolved O_2_ to the bacteroids in order to support high respiration associated with nitrogenase activity [71]. Sesbania flooded nodules had a somewhat lower concentration of leghemoglobin when compared to that of non-flooded nodules. In contrast, an earlier study found no differences in leghemoglobin concentration between non-flooded and flooded *Neptunia* nodules [72]. James and Crawford (1998) assayed leghemoglobin in flooded *Lotus* nodules and concluded that in the tolerant species, *L. uliginosus*, leghemoglobin levels were lower than in the non-tolerant species, *L. corniculatus*, because O_2_ supply to the nodules was at least adequate in the tolerant species owing to the suite of adaptations to flooding that it possesses [32]. The concentration of leghemoglobin in Sesbania flooded nodules appears to be sufficient to maintain adequate O_2_ supply to sustain nitrogen fixation. This fact is reflected in the plants ability to thrive in flooded conditions and produce more biomass.

## 4. Materials and Methods

*Sesbania herbacea* seeds were purchased from Adams-Briscoe Seed Company, Jackson, GA, USA. Sesbania seeds (about 25–30 seeds per meter of row) were planted in the non-cultivated part of a rice pan at the University of Missouri Delta Center, Portageville, MO, USA. Sesbania seeds were planted in two field plots (approximately 0.5 hectare) during late May. After the seedlings emerged, one plot was flooded (water level maintained 5 to 10 cm deep) and the other plot was left dry. Flooded field conditions were maintained throughout the growth until the seeds were harvested during September. Sixty days after planting, Sesbania plants were randomly selected from each plot, roots were washed in running water, and root nodules were collected and stored at −80 °C until used. For anatomical studies, freshly harvested nodules were immediately fixed in chemical fixatives and processed for light and electron microscopy, as described later. Fully grown mature plants harvested from a 2.5 m × 2.5 m sample area was used for the measurement of biomass, pod numbers, and stem diameter. Total nitrogen/protein content of Sesbania seeds was measured using the Leco model FP-428 nitrogen analyzer (LECO Corporation, St Joseph, MI, USA).

### 4.1. Greenhouse Experiments

Sesbania seeds were surface-sterilized in 50% bleach (2.5% final NaClO) for 5 min, rinsed extensively in distilled water, and germinated on 1% water agar plates at 30 °C for 3 days. The roots of germinated 3-day-old seedlings were placed in 2 mL Eppendorf tubes containing 1 × 10^8^ cells/mL of *Rhizobium huautlense* for 2 min. Following the inoculation, the seedlings were transferred to small pots filled with sterile vermiculite and placed in a growth chamber that was maintained at a constant temperature of 28 °C. The light intensity in the growth chamber was maintained at 500 μmol·m^2−1^·s^−1^ with a 14 h light period. Pots with and without holes at the bottom were used for imposing non-flooding and flooding treatments, respectively. To create flooding condition, pots were filled with water so that at least 5 cm of the shoot system was constantly immersed in water. Plant height, shoot fresh weight, number of nodules, and nitrogenase activity were measured at the end of 30 days growth. The nitrogen fixation rate measured by acetylene reduction assay was performed by the method of Schwinghamer [73] on whole root systems containing intact nodules, after removal of the vegetative portion of the plant. Six individual root systems were analyzed for nitrogenase activity for each treatment, over a 12 min time course with air samples taken every 2 min. Activity was collected as the initial velocity (µmol·min^−1^) of ethylene produced and calculated to mmol h^−1^ mg^−1^ of fresh weight of all nodules within the sealed assay vial.

### 4.2. Embedment of Sesbania Nodules in Paraffin

Field grown Sesbania nodules grown under non-flooded and flooded conditions were fixed in 50% ethanol, 5% glacial acetic acid and 10% formaldehyde (FAA) and processed for embedment in paraffin, as described earlier [74]. Nodule sections (10-μm thick) were cut with a microtome and stained with hematoxylin and eosin.

### 4.3. Light and Transmission Electron Microscopy

Samples for light and transmission electron microscopy were processed essentially, as described earlier [74]. For light microscopy observations, the nodule sections were stained with 1% toluidine blue and examined with bright-field optics. For electron microscopy, thin sections were stained with 0.5% uranyl acetate and 0.4% lead citrate and examined with a 1200 EX transmission electron microscope (JEOL, Tokyo, Japan) at 80 kV.

### 4.4. Protein Isolation, 1-D SDS-PAGE, and Immunoblot Analysis

Total proteins were extracted from 100 mg of frozen nodules with 1 mL of SDS sample buffer (2% SDS, 60 mM Tris-HCl, pH 6.8, 5% β-mecaptoethanol). The samples were boiled at 100 °C for 5 min followed by centrifugation at 15800× *g* for 5 min. The resulting supernatant was treated as the total nodule protein fraction and was resolved by 15% SDS-PAGE gels using a Hoeffer SE 250 mini-vertical electrophoresis apparatus (GE Healthcare, Pittsburgh, PA, USA). Resolved proteins were visualized by staining overnight with Coomassie Brilliant Blue. Western blot analysis was performed as described earlier [75]. Polyclonal antibodies raised against leghemoglobin, malate dehydrogenase, AspAT-1, and AspAT-2 were obtained from Dr. Carol Vance (USDA-ARS). These antibodies were used at 1:5000 dilution. Sesbania nodule proteins specifically reacting against individual antibodies were detected with the aid of an enhanced chemiluminescent substrate (SuperSignal West Pico kit, Pierce, Rockford, Il, USA).

### 4.5. Protein Isolation, 2-D SDS-PAGE and Image Analysis

Two-dimensional gel electrophoresis of Sesbania nodule bacteroid proteins were performed as described earlier [76]. Coomassie stained gels were destained with multiple changes of distilled water to remove background. The gels were imaged with an Epson Perfection V700 scanner controlled through Adobe Photoshop. Images were analyzed for proteome differences using Delta2D (v. 4.4.1) image analysis software.

### 4.6. MALDI-TOF/MS

Sesbania nodule bacteroid proteins showing enhanced accumulation under flooded conditions were excised from the gel and digested with trypsin. Digested peptides were subjected to MALDI-TOF MS analysis for protein identification, as described previously [77]. The MS/MS results were analyzed using Mascot version 2.3.02 (Matrix Science, Boston, MA, USA). The peptide sequences generated from this analysis were subjected to BLAST search against the National Center for Biotechnology Information non-redundant (NCBInr) protein database using the appropriate taxonomy filter (prokaryotes).

### 4.7. Extraction of Secondary Metabolites

Ground root nodule samples (100 mg) were extracted with 1 mL of methanol: water (80: 20 v/v) using ultrasonic assisted extraction for 10 min [78]. The extracts were centrifuged at 8000xg for a period of 10 min and the supernatant was collected into a separate tube. This extraction procedure was repeated two additional times using the same solvent mixture and the pooled supernatants were evaporated to dryness using a SpeedVac. Finally, the dried pellets were re-dissolved in 80% methanol, filtered through polyvinylidene difluoride (PVDF) syringe filters (pore size 0.45 μm, National Scientific Company, Duluth, GA, USA). The filtered extracts were analyzed by LC-MS.

### 4.8. Amino Acids Extraction

The amino acid extraction and analysis was achieved using a slightly modified procedure as described by Redruello et al. [79]. Ground root nodules (50 ± 1mg) were extracted with 2.0 mL 0.1N HCl containing 0.2% TPDA (thiodipropionic acid). The mixture was placed in an ultrasonication bath (for a period of 15 minutes. The collected supernatant was filtered into a 2 mL microfuge tube. Supernatant aliquot (500 µL) was derivatized by mixing with 175 µL of 1 M borate buffer (pH 9.0), 75 µL MeOH, and 15 µL DEEMM. The mixture was sonicated at ambient temperature for 45 mins followed by incubation at 70 °C for 2 h. The derivatized amino acids mixture was filtered and analyzed by LC-MS.

### 4.9. Soluble Sugars Extraction

The soluble sugars were extracted using a previously published procedure [80]. In brief, 20 ± 1 mg of ground root nodules were extracted in 5 mL DI H2O using ultrasonic assisted extraction for 10 min. The mixture was centrifuged at 8000× *g* for 15 min. The collected supernatant was filtered and diluted prior to IC-MS analysis.

### 4.10. LC-MS Analysis for Secondary Metabolites

Secondary metabolites were analyzed using Agilent 1290 LC (Agilent Technology, Santa Clara, CA, USA) coupled with Thermoscientific MSQ Plus MS system, Waltham, MA, USA) with Luna 5 µ C18 (2) 100 Å column (150 × 4.6 mm) as described in our earlier publication [78]. Water (A) and acetonitrile (B) contains 0.1% formic acid (v/v) served as mobile phase. The initial gradient of the mobile phase was maintained at 10% acetonitrile for 5 min, followed by increase to 40% at 30 min, 60% at 40 min, and 90% at 50 min. This condition was maintained for another 5 min followed by a decrease to 10% at 60 min. Twenty microliters of the sample was injected and the flow rate was maintained at 0.7 mL·min^−1^. Mass spectra were obtained using electrospray ionization in negative modes within a range of 100–1000 m/z and the cone voltage was 90 V; probe temperature was 500. All extraction and analyses were carried out in triplicate.

### 4.11. LC-MS Analysis for Amino Acids

For the amino acid analysis, 25 mM ammonium acetate buffer (pH 6.7) containing 0.02% sodium azide (A), and methanol: water (8:2 v/v) (B) was served as mobile phase. The gradient conditions are as follows: Between 0 to 40 min, the gradient used was 90% (A) and 10% (B). At 42 min, the gradient was changed to 70% A and 30% B. After 77 min, gradient was changed to 100% B. After 82 mins, the gradient was set back to initial conditions (90% (A) and 10% (B)). Electrospray ionization MS was performed in the negative (−) and positive (+) ion mode over a range of 100–1000 m/z. The operating parameters were as follows: Ion source temperature, 300 °C; cone gas flow, 50 L·h^−1^; desolvation gas flow, 600 L·h^−1^; capillary voltage, 2.8 kV; and cone voltage, up to 35 V.

### 4.12. IC-MS Analysis for Sugarslog10

IC-MS analysis was carried out in a Dionex ICS 5000 MSQ (Thermoscientific) system equipped with Dionex AS-AP auto-sampler and Dionex ICS 5000 EG-5 eluent generator. ICS 5000 single pump was used for the mobile phase and the metabolites were separated by an IonPac PA20 (3 × 150 mm i.d.) column equipped with IonPac guard column [80]. Injection volume was 100 µL with the slit ratio of 50:50. The MS analysis was done with the electron spray ionization (ESI) Surveyor MSQ system from Thermoscientific. Calibration curve with individual and mixed sugar samples at different concentrations was developed prior to bean sample analysis. For analysis of sugars, the retention time of the peaks were compared with individual standards.

### 4.13. Data Processing and Multivariate Analysis

The peak area of all the samples were converted in to log_10_ value for comparison. In the case of amino acids and sugars, the quantified metabolites (targeted analysis) were used for the multivariate analysis. SIMCA software 13.0 (Umetrics, Umeå, Sweden) was used for the analysis. Partial least-squares-discriminate analysis (PLS-DA) model was carried out using auto-scaled and log-transformed data and the clustering pattern was viewed. The key metabolites were compared by plotting box-whisker plot using STATISTICA, V7 software (TIBCO Software Inc., Palo Alto, CA, USA).

### 4.14. Enzyme Analysis

Nodules (250 mg) were placed into a pre-chilled mortar and pestle with a small amount of acid washed sand and 5 mL of cold MPS (5 mM MgCl_2_, 50 mM potassium phosphate buffer, pH 7.2, 17% *w/v* sucrose) with protease inhibitor cocktail. Mixture was ground into a liquid and placed on ice. Solution was centrifuged at 400× *g* for 10 min at 5 °C to remove plant material. Supernatant was placed into a fresh tube and centrifuged at 8000× *g* for 20 min at 5 °C to pellet bacteroids. Supernatant was placed into a fresh tube (nodule plant cytosol) for use in enzyme assays. Protein quantification was performed using the method of Bradford. Typically, 10–50 µg of protein was used for each enzyme assay. All assay volumes were 1 mL and performed at 25 °C using a Varian Bio50 spectrophotometer using real-time kinetics program. Initial linear velocity was used to calculate product formation.

Alcohol dehydrogenase (ADH, EC 1.1.1.1) was assayed in the direction of ethanol production and contained 100 mM Tris-Cl, pH 8, 0.5 mM ZnCl_2_, 5 mM acetaldehyde, 0.2 mM NADH, and monitored for a decrease in absorbance of NADH at A_340_. No activity was found in the direction of acetaldehyde production. Alanine aminotransferase (AlaAT, EC 2.6.1.2) was assayed in the direction of pyruvate production and contained 100 mM Tris-Cl, pH 8.0, 5 mM NH_4_Cl, 10 µg/mL pyridoxal 5′-phosphate, 5 mM 2-oxoglutarate, 10 mM l-alanine, 5 U of lactate dehydrogenase, 0.2 mM NADH, and monitored for a decrease in absorbance of NADH at A_340_. Aspartate aminotransferase (AspAT, EC 2.6.1.1) was assayed in the direction of oxaloacetate production and contained 100 mM Tris-Cl, pH 8.0, 10 µg/mL pyridoxal 5′-phosphate, 5 mM 2-oxoglutarate, 10 mM l-aspartate, 5 U of malate dehydrogenase, 0.2 mM NADH, and monitored for a decrease in absorbance of NADH at A_340_. Glutamate dehydrogenase (GDH, EC 1.4.1.3) was assayed in the direction of glutamate production and contained 100 mM Tris HCl, pH 8.0, 100 mM NH_4_Cl, 10 mM 2-oxoglutarate, 0.2 mM NADH, and monitored for a decrease in absorbance of NADH at A_340_. Malate dehydrogenase (MDH, EC 1.1.1.37) assay contained 100 mM Tris-Cl, pH 8.0, 10 mM oxaloacetate, 10 µg/mL pyridoxal 5′-phosphate, 0.2 mM NADH, and monitored for a decrease in absorbance of NADH at A_340_. PEP carboxylase (PEPC, EC 4.1.1.31) assay contained 100 mM Tris-Cl, pH 8.0, 5 mM phosphoenolpyruvate, 5 mM MgSO_4_, 5 mM NaHCO_3_, 5 U of lactate dehydrogenase, 5 U of malate dehydrogenase, 0.2 mM NADH, and monitored for a decrease in NADH at A_340_.

## Figures and Tables

**Table 1:** Effect of flooding on various parameters of *Sesbania herbacea*.

Parameter	Treatment (Location)	
	Non-Flooded (Field)	Flooded (Field)
Biomass (dry weight, kg·ha^−1^)	2730 ± 541	7021 ± 633
Plant height (cm; *n* = 24)	199.39 ± 35.41	361.63 ± 29.99
Basal stem diameter (mm; *n* = 24)	12.29 ± 2.14	18.92 ± 3.49
Pods/plant (total number; *n* = 24)	104 ± 43	109 ± 30
Seed protein content (%; *n* = 8)	35.05 ± 1.17	37.94 ± 4.40
	**Non-Flooded (Greenhouse)**	**Flooded (Greenhouse)**
Number of nodules/plant (*n* = 15)	10 ± 3	70 ± 19
Plant height (cm; *n* = 15)	17.91 ± 2.61	27.02 ± 5.53
Nodule fresh weight (grams/nodule; *n* = 15)	0.017 ± 0.006	0.020 ± 0.007
Nitrogenase activity (C_2_H_4_ mmol·h^−1^·mg^−1^; acetylene reduction, *n* = 6)	5.25 ± 0.45	4.90 ± 0.90

* Field data were collected in six smaller (2.5 m × 2.5 m) plots scattered throughout the larger 0.5 hectare plot. Data on yield and biomass were estimated for the entire plot using the average of all six smaller plots. Plant height, stem diameter, and pods/plant data were collected by harvesting four plants from each smaller plot. Seed protein content data were from eight separate analyses using combined ground seed powder, from field grown Sesbania throughout each of the six smaller plots. Greenhouse (growth chamber) data were from 15 individual plants per treatment. Measurements were carried out on 40-day-old plants.

**Table 2:** Proteins identified from flooded Sesbania nodule bacteroids after 2D SDS-PAGE separation and peptide mass fingerprinting *.

#	Protein ID *	MOWSE	Peptides Matched	% C	pI E/T	M_r_ E/T	MS-Fit NCBIprot: Acc. #
**1**	TldD/PmbA family (*Neorhizobium huautlense*)	275	3	8	5.00/4.84	48,000/47,343	1::WP_105374151.1
**2**	No match						
**3**	Elongation factor Tu (*Neorhizobium huautlense*)	453	7	17	5.60/5.29	43,000/42,686	1::WP_105374387.1
**4**	Branched chain amino acid ABC transporter (*Neorhizobium huautlense*)	781	10	26	5.30/5.34	41,000/39,196	1::WP_105372985.1
**5**	Phosphonate ABC transporter (*Neorhizobium huautlense*)	883	8	37	5.50/5.34	36,000/36,238	1::WP_105370531.1
**6**	Nitrogenase iron protein (*Neorhizobium huautlense*)	938	12	39	5.10/4.83	33,000/31,945	1::WP_105375136.1
**7**	Sugar ABC transporter (*Neorhizobium huautlense*)	509	6	29	5.60/5.59	33,000/35,397	1::WP_105371152.1
**8**	No match						
**9**	No match						
**10**	HSP20/alpha crystalline family (*Neorhizobium huautlense*)	158	2	12	6.10/5.87	28,000/19,836	1::WP_105372823.1
**11**	Fructose-6-PO_4_ aldolase (*Mesorhizobium* sp.)	213	3	15	6.15/5.46	25,000/23,332	1::WP_041004472.1
**12**	Ferritin-like domain-containing (*Neorhizobium huautlense*)	346	4	27	5.90/4.92	23,000/18,185	1::WP_105371465.1
**13**	Peroxiredoxin (*Neorhizobium huautlense*)	250	3	20	6.15/5.61	23,000/20,344	1::WP_105375072.1
**14**	sn-glycerol-3-PO4 ABC trans-porter, UgpB (*Neorhizobium huautlense*)	750	11	28	8.00/8.17	43,000/48,045	1::WP_105370415.1
**15**	sn-glycerol-3-PO4 ABC trans-porter, UgpB (*Neorhizobium huautlense*)	646	9	18	8.80/8.17	43,000/48,045	1::WP_105370415.1
**16**	Phasin (*Neorhizobium huautlense*)	390	21	45	7.50/6.75	16,000/16,394	1::WP_105374889.1

* Spot identification #’s correspond to those proteins labeled in Figure 6. Isoelectric point (pI) and molecular weight (M_r_) values are given as experimental/theoretical values. MOWSE scores represent those searches performed via Mascot at 50 ppm error. Peptides matched, %C (coverage), and accession numbers are from MASCOT searches confined to microorganism databases within the National Center for Biotechnological Information (NCBI non-redundant) database. Protein spots 14, 15, and 16 were taken from a 2D SDS-PAGE pI 3-10 isoelectric focused gel of flooded bacteroid proteins that were not present in non-flooded bacteroids (gel image not shown).

## Supplementary Materials

Supplementary materials can be found at https://www.mdpi.com/1422-0067/20/8/1824/s1.

## Abbreviations

1-D	One-dimensional
2-D	Two-dimensional
NH3	Ammonia
ATP	Adenosine triphosphate
MDH	Malate dehydrogenase
ADH	Alcohol dehydrogenase
GDH	Glutamate dehydrogenase
PEPC	Phosphenolpyruvate carboxylase
AspAT	Aspartate aminotransferase
AlaAT	Alanine aminotransferase
GABA	Gamma-Amino Butyric acid
LC-MS	Liquid chromatography–mass spectrometry
IC-MS	Ion chromatography–mass spectrometry
MALDI-TOF/MS	Matrix-Assisted Laser Desorption Ionization-Time of Flight/MS
TEM	Transmission electron microscopy
PBM	Peribacteroid membrane
DAI	Days after inoculation
PHB	Poly β-hydroxybutyrate
EPS	Exopolysaccharides
LPS	Lipopolysaccharides
