# Supplementary material for: Biochemical and Anatomical Investigation of Sesbania herbacea (Mill.) McVaugh Nodules Grown under Flooded and Non-Flooded Conditions
Source: Int J Mol Sci. 2019 Apr 12;20(8):1824. doi: 10.3390/ijms20081824 (PMC6514687; doi:10.3390/ijms20081824)
Supplement: Supplementary file 1 [file ijms-20-01824-s001.pdf]

Supplemental Table 1. Particulars of the metabolites identified by LC-MS and IC-MS in flooded and non-flooded nodules.

#	Time	p value	Method (metabolites)	Name	Reference
1	30.33	0.0139	LC-MS (Secondary)	Ononin	MS and ref
2	33.04	0.0209	LC-MS (Secondary)	Glycetein	MS and ref
3	45.29	0.0209	LC-MS (Secondary)	Formonetin	MS and ref
4	3.87	0.0833	LC-MS (Primary)	Aspartic Acid	Standard
5	5.42	0.1489	LC-MS (Primary)	Glutamic Acid	Standard
6	8.38	0.0209	LC-MS (Primary)	Asparagine	Standard
7	9.04	0.0209	LC-MS (Primary)	Serine	Standard
8	9.51	0.7728	LC-MS (Primary)	Histidine	Standard
9	9.77	0.1489	LC-MS (Primary)	Glutamine	Standard
10	13.35	0.0433	LC-MS (Primary)	Glycine	Standard
11	15.75	0.0209	LC-MS (Primary)	Threonine	Standard
12	27.26	0.0433	LC-MS (Primary)	L -Alanine	Standard
13	31.11	0.0209	LC-MS (Primary)	GABA	Standard
14	45.33	0.7726	LC-MS (Primary)	Tyrosine	Standard
15	48.04	0.0433	LC-MS (Primary)	Valine	Standard
16	53.49	0.0433	LC-MS (Primary)	Tryptophan	Standard
17	54.27	0.0209	LC-MS (Primary)	Isoleucine	Standard
18	56.19	0.1489	LC-MS (Primary)	Leucine	Standard
				Phenylalanine	
19	77.96	0.0433	LC-MS (Primary)	Lysine	Standard
20	10.29	0.0209	IC-MS (Primary)	Glucose	Standard
21	11.43	0.0209	IC-MS (Primary)	Sucrose	Standard
22	12.15	0.2482	IC-MS (Primary)	Fructose	Standard
23	13.40	0.1489	IC-MS (Primary)	Melibiose	Standard
24	35.41	0.0209	IC-MS (Primary)	Maltose	Standard

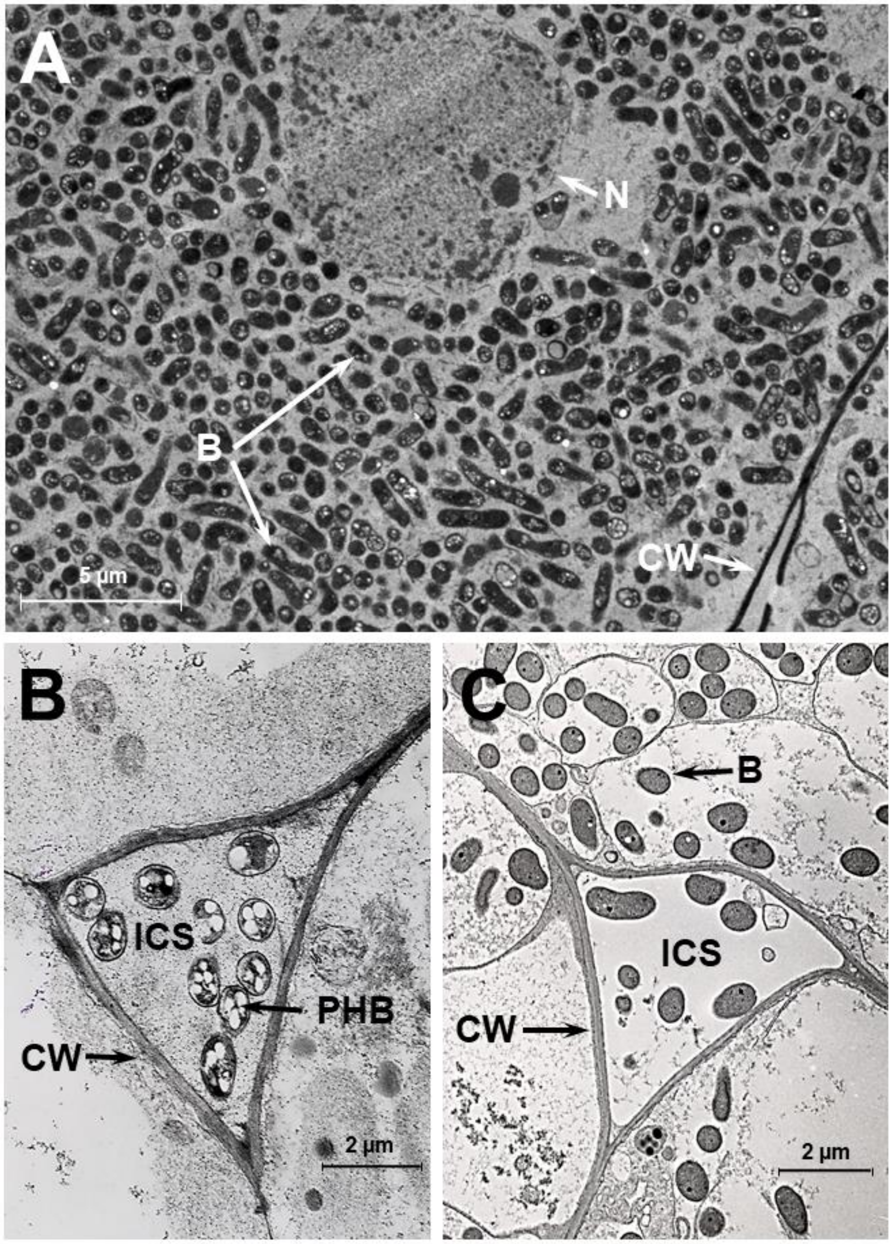

Supplemental Figure 1. Transmission electron micrographs of Sesbania nodules. A. Low magnification view of Sesbania flooded nodule cell harboring bacteroids with prominent polyhydroxybutyrate crystals. B. Rhizobia were also seen in the intercellular space. C. Electron micrograph of non-flooded Sesbania nodule cell reveal the presence of numerous bacteroids. Note the absence of polyhydroxybutyrate crystals in these bacteroids. B, bacteroid; CW, cell wall; ICS, inter cellular space; N, nucleus; PHB, polyhydroxybutyrate.

1A

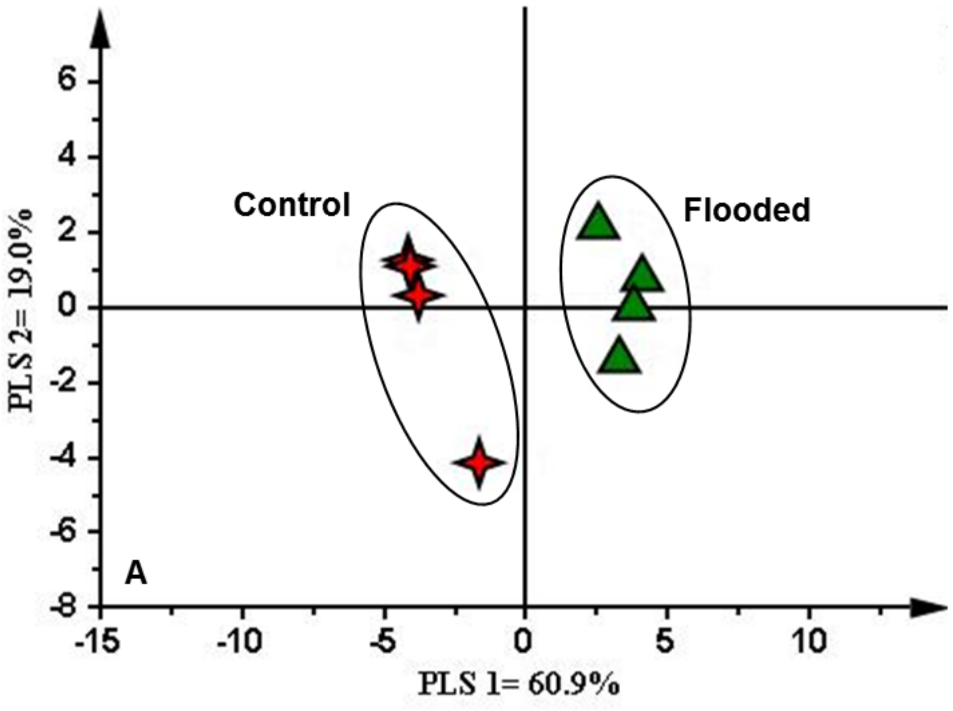

1B

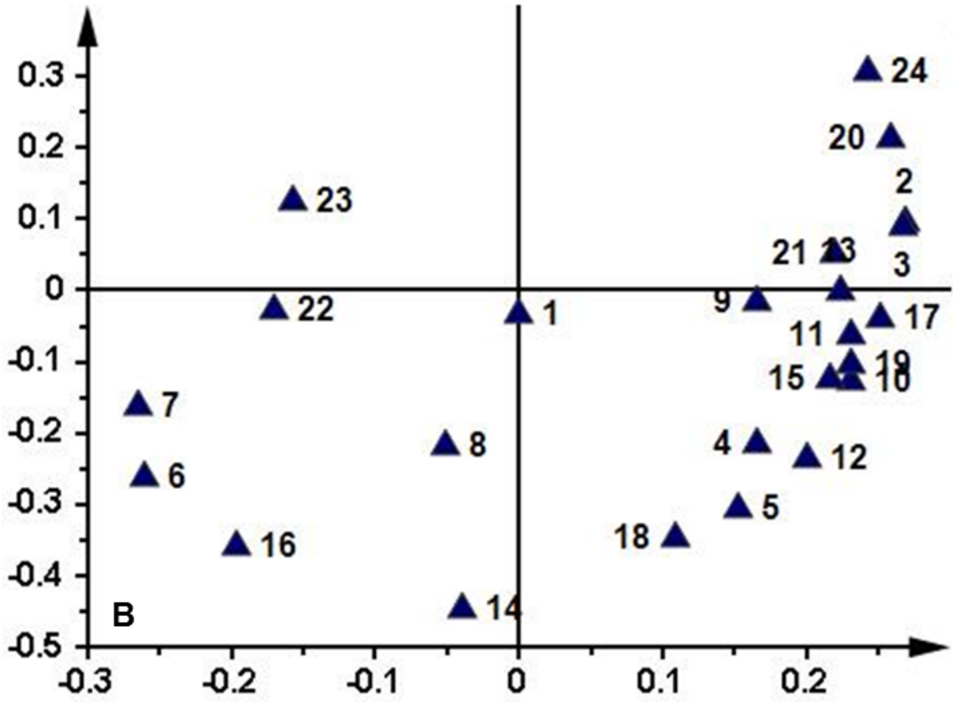

Supplemental Figure 2. Metabolic variation between non-flooded and flooded Sesbania nodules. (1A). PLS-DA score plot. (1B). PLS-DA loading plot. Metabolite # corresponds to those listed in Supplemental Table 1.
